# ATP and Formyl Peptides Facilitate Chemoattractant Leukotriene-B4 Synthesis and Drive Calcium Fluxes, Which May Contribute to Neutrophil Swarming at Sites of Cell Damage and Pathogens Invasion

**DOI:** 10.3390/biomedicines12061184

**Published:** 2024-05-27

**Authors:** Ekaterina A. Golenkina, Galina M. Viryasova, Svetlana I. Galkina, Iuliia V. Iakushkina, Tatjana V. Gaponova, Yulia M. Romanova, Galina F. Sud’ina

**Affiliations:** 1 Belozersky Institute of Physico-Chemical Biology, Lomonosov Moscow State University, 119234 Moscow, Russia; golyesha@mail.ru (E.A.G.); galina.viryasova@yandex.ru (G.M.V.); galkina@genebee.msu.ru (S.I.G.); dddd80486@gmail.com (I.V.I.); 2 National Research Center for Hematology, Russia Federation Ministry of Public Health, 125167 Moscow, Russia; gaponova.tatj@yandex.ru; 3 Department of Genetics and Molecular Biology, Gamaleya National Research Centre of Epidemiology and Microbiology, 123098 Moscow, Russia; genes2007@yandex.ru

**Keywords:** neutrophil, *Salmonella typhimurium*, leukotriene B4, ATP, intracellular calcium, neutrophil swarming

## Abstract

Here, we demonstrate that human neutrophil interaction with the bacterium *Salmonella typhimurium* fuels leukotriene B4 synthesis induced by the chemoattractant fMLP. In this work, we found that extracellular ATP (eATP), the amount of which increases sharply during tissue damage, can effectively regulate fMLP-induced leukotriene B4 synthesis. The vector of influence strongly depends on the particular stage of sequential stimulation of neutrophils by bacteria and on the stage at which fMLP purinergic signaling occurs. Activation of 5-lipoxygenase (5-LOX), key enzyme of leukotriene biosynthesis, depends on an increase in the cytosolic concentration of Ca^2+^. We demonstrate that eATP treatment prior to fMLP, by markedly reducing the amplitude of the fMLP-induced Ca^2+^ transient jump, inhibits leukotriene synthesis. At the same time, when added with or shortly after fMLP, eATP effectively potentiates arachidonic acid metabolism, including by Ca^2+^ fluxes stimulation. Flufenamic acid, glibenclamide, and calmodulin antagonist R24571, all of which block calcium signaling in different ways, all suppressed 5-LOX product synthesis in our experimental model, indicating the dominance of calcium-mediated mechanisms in eATP regulatory potential. Investigation into the adhesive properties of neutrophils revealed the formation of cell clusters when adding fMLP to neutrophils exposed to the bacterium *Salmonella typhimurium*. eATP added simultaneously with fMLP supported neutrophil polarization and clustering. A cell-derived chemoattractant such as leukotriene B4 plays a crucial role in the recruitment of additional neutrophils to the foci of tissue damage or pathogen invasion, and eATP, through the dynamics of changes in [Ca^2+^]_i_, plays an important decisive role in fMLP-induced leukotrienes synthesis during neutrophil interactions with the bacterium *Salmonella typhimurium*.

## 1. Introduction

Neutrophils (polymorphonuclear leukocytes, PMNL) are the most numerous leukocytes circulating in the blood; these are the first immune cells recruited by invading pathogens or damaged cells [1,2]. Chemoattractants elicited by pathogens or damaged cells form a concentration gradient that determines the direction of PMNLs extravasation and tissue migration. Extremely high chemotactic potential is possessed by the breakdown products of bacterial and mitochondrial proteins—formylated methionine bearing peptides (N-formyl peptides). Formylated peptide receptors (FPRs), namely FPR1 and FPR2, are abundantly expressed by neutrophils [3]. It is the signaling induced by formylated peptides of bacterial origin that ensures the early recruitment of neutrophils to the sites of pathogen invasion [4]. Among FPR1 agonists, N-formyl-methionyl-leucyl-phenylalanine (fMLP), a prototypic representative of bacterial chemotactic factor, has been identified as the most potent one [5]. During unregulated cell death, mitochondrial N-formyl peptides are released into the extracellular environment and act as chemoattractants for clearance of dead cells [6].

Extracellular ATP (eATP) represents damage-associated molecular patterns (DAMPs) released from damaged cells [7]. It is likely that formyl peptides, together with ATP, organize complex processes that guide phagocytes during their moving to and final meetings with target cells at the sites of inflammation.

In mammalian cells, ATP is formed by oxidative phosphorylation or glycolysis and stored at cells at intracellular concentrations of ~5 mM. In healthy tissues, the extracellular ATP concentration is very low, ~10 nM, due to plasma membrane-anchored ectonucleotidases [8]. When the tissue is damaged, cell death, accompanied by disruption of membrane integrity, leads to a sharp uncontrolled ATP release. Channel-mediated and vesicle-dependent ATP emission are controlled mechanisms for increasing ATP amount in the extracellular milieu [9]. eATP increases dramatically in pathological conditions, in particular during inflammation [10,11]. This small metabolite emission is considered to be one of the biomarkers of immunogenic cell death [12]. DAMPs released into the intercellular space during cellular stress or tissue damage coordinate the severity of the immune response. Extracellular ATP serves as a “find-me” signal to attract phagocytes that mediate the clearance of dying cells and necrotic bodies [13,14].

ATP and its metabolites act through two different types of purinergic receptors: nucleosides, such as adenosine, are recognized by P1 receptors, while P2 receptors are sensitive to nucleotides. Depending on the mechanism of action, ionotropic and metabotropic P2 receptors (P2XR and P2YR, respectively) are distinguished [7]. The interaction of ATP as well as UTP released from necrotic cells with P2Y2 receptors appears to be important for the detection of necrotic cells by neutrophils and macrophages [15]. ATP release and concomitant P2Y2 receptor signaling promote phagocytic clearance of apoptotic cells or bacteria by macrophages and neutrophils [13]. P2X receptors function as ligand-gated ion channels [16,17] that facilitate the influx of extracellular calcium [18]. ATP is a native agonist for all seven P2X receptors [19]. P2X7R is constitutively expressed on the cell surface of murine and human neutrophils [20]. ATP, but not UTP, binding to P2X7R facilitates bacterial clearance by activating the NLRP3 inflammasome and IL-1β secretion [20,21].

Increased eATP concentration plays an important role in the immune response to intracellular pathogens. Thus, it is purinergic signaling that activates human macrophages to kill Mycobacteria [22]. Chlamydial infection of neutrophils and epithelial cells produce elevated levels of extracellular ATP, which promotes macrophages stimulation [23]. Extracellular nucleotides have been shown to potentiate the killing of *Leishmania donovani* by neutrophils. Exposure to ATP and UTP leads to activation, enhanced CD11b expression, and strongly intensified *Leishmania*-induced ROS [24].

During bacterial infection, formyl peptides receptors facilitate the killing and clearance of microbes [25,26]. The interaction of “pioneer” neutrophils with pathogen or danger signal from damaged tissue results in leukotriene synthesis, and leukotriene B4 (LTB4) release leads to exponential accumulation or swarming of neutrophils in the lesion [27]. In sterile injuries, ATP plays a role in neutrophil communication and clustering [28]. LTB4 synthesis has been shown to be triggered by a sustained calcium flux upon contact with the necrotic tissue [28]. Cx43 hemichannels, which are mediators of active ATP release, are also required for maximal wound defense from bacterial invasion [28], while ATP signaling clearly modulates the level of leukotriene B4 in neutrophils exposed to necrotic cells (DAMPs) [28].

Neutrophils accumulate in the injured tissue and move in ‘swarms’ due to the coordinated release of a strong chemoattractant, leukotriene B4. Primary signals of injury/infection have a relatively short range of action, and activated neutrophils release chemoattractants such as leukotriene B4 for positive-feedback-based recruitment of additional neutrophils [27,29]. End-target chemoattractants formyl peptides collaborate with intermediate-target chemoattractants (lipid leukotriene B4 and chemokines, e.g., CXCL8) to navigate neutrophil trafficking [30]. This process must be tightly regulated to limit collateral damage [31]. The effects of ATP have been studied in a model system, the zebrafish [28]. To understand neutrophil swarming, it is important to study these processes on human neutrophils because of known differences in primary human neutrophil behaviors compared to model systems [32,33]. Sensing of eATP is important for induction of LTB4 synthesis by damage and sustained calcium fluxes upon contact with necrotic tissue [28]. Less is known about the regulation by ATP of leukotriene synthesis in human neutrophil interaction with pathogens. Recent studies have identified leukotriene B4 as the “unique intercellular communication signal between neutrophils” for the recruitment of cells during swarming. In our study, we found that ATP and formyl peptides tightly cooperate and facilitate the chemoattractant LTB4 synthesis in human neutrophil interaction with the gram-negative bacterium *Salmonella typhimurium.*

## 2. Materials and Methods

### 2.1. Materials

ATP was obtained from Biomol (Hamburg, Germany). Formyl-Met-Leu-Phe (fMLP), fibrinogen from human plasma, flufenamic acid, glybenclamide, calmodulin inhibitor R 24571, adenosine, Hank’s balanced salt solution with calcium and magnesium but without Phenol Red and sodium hydrogen carbonate (HBSS), and Dulbecco’s PBS with magnesium but without calcium (D-PBS) were purchased from Sigma-Aldrich (Steinheim, Germany). Hoechst was obtained from Molecular Probes (Mo Bi Tec, Göttingen, Germany). Acetoxymethyl-ester (AM) conjugated fura-2 was purchased from Thermo Fisher Scientific (Waltham, MA, USA).

Bacteria (*S. typhimurium* IE 147 strain) (S_147_) were obtained from the Collection of Gamaleya National Research Center of Epidemiology and Microbiology (Moscow, Russia). Bacteria were grown in Luria–Bertani broth to a concentration of 1 × 10^9^ colony-forming units (CFU)/mL. Non-opsonized and opsonized bacteria were used in this work.

### 2.2. PMN Leukocyte Isolation

PMNLs were isolated from freshly drawn citrate-anticoagulated blood donated by healthy adult volunteers of either sex. Experimental and the subject consent procedures were approved by the Bioethics Committee of the Lomonosov Moscow State University, Application # 6-h, version 3, Bioethics Commission meeting # 131-d held on 31 May 2021. Leukocyte-rich plasma was prepared by dextran T-500 sedimentation of erythrocytes at room temperature, and PMNLs were obtained as described [34]. Neutrophils (96–97% purity, 98–99% viability as established by trypan blue staining) were suspended at 2 × 10^7^ cells/mL D-PBS containing 1 mg/mL glucose and stored at room temperature.

### 2.3. Incubations for 5-LOX Product Synthesis in Cells

PMNLs ((1.2–1.5) × 10^7^/6 mL HBSS/Hepes) were placed in a CO_2_ incubator at 37 °C for 10 min, then bacteria or reagents were added, as indicated. The incubations were stopped by adding an equal volume of methanol (−18 °C) with 90 ng prostaglandin B2 as internal standard. Major metabolites of 5-LOX, 5S,12R-dihydroxy-6,14-cis-8,10-trans-eicosatetraenoic acid (LTB4), iso-LTB4 (5S,12SR-alltrans-diHETE) (t-LTB4), ω-OH-LTB4, ω-COOH-LTB4, and 5Shydroxy-6-trans-8,11,14-cis-eicosatetraenoic acid (5-HETE) were identified as previously described [35].

### 2.4. Calcium Flux Assay

To detect changes in intracellular calcium concentration ([Ca^2+^]_i_), the ratiometric calcium-sensitive fluorescent dye fura-2 AM was used. The manufacturer’s instructions were partially adapted to work with neutrophils. Briefly, isolated PMNLs (10^7^ cells/mL) were incubated with 1 µM fura-2 AM in Ca^2+^-free Dulbecco’s PBS for 30 min at 37 °C. Then, cells were pelleted (200 g, 10 min), washed once with PBS, and resuspended in Dulbecco’s PBS. Immediately before the experimental procedure, labeled cells were resuspend in HBSS/HEPES medium, seeded in fibrinogen-coated black 96-well F-bottom plates, and treated according to the experimental design at 37 °C in 5% CO_2_. Reagent injectors integrated into the reader platform were used for stimuli addition. Changes in fluorescence emitted at 510 nm were measured when exited by both 380 nm (for Ca^2+^-free dye) and 335 nm (for Ca^2+^-bound dye) every 0.6 s. Manipulations were performed on a CLARIOstar multimode microplate reader (BMG Labtech, Cary, NC, USA) and the MARS data analysis software package version 3.30 from BMG Labtech was used to process the data obtained. [Ca^2+^]_i_ shifts were judged by changes in the ratio of fluorescence intensities produced by excitation at two wavelengths. Data were quantified using areas under the kinetic curves (AUC) above the baseline.

### 2.5. PMNLs Adhesion Assessment

PMNLs substrate adhesion was assessed by colorimetric detection of 2,3-diaminophenazine formed in the myeloperoxidase-catalyzed reaction of o-phenylenediamine (OPD) with H_2_O_2_ [36,37]. PMNLs (2 × 10^5^ cells/sample) were seeded into the fibrinogen-coated wells of 96-well plates with pre-warmed HBSS/HEPES and incubated in accordance with the experimental protocol. At the end of treatment, free and poorly attached cells were removed by washing twice with warm PBS. A total of 5.5 mM OPD and 4 mM H_2_O_2_ in permeabilizing buffer (67 mM Na_2_HPO_4_, 35 mM citric acid and 0.1% Triton X-100) was added to the wells and, after 5 min, the reaction was stopped with 1 M H_2_SO_4_. To construct calibration curves allowing to determine the percentage of attached neutrophils, the reaction was also carried out in wells containing known numbers of cells. The optical density (490 nm) of each well was determined with CLARIOstar multimode microplate reader.

### 2.6. Microscopy

PMNLs were seeded into fibrinogen-coated confocal dishes with pre-warmed HBSS/HEPES (10^6^ cells/dish). At the end of the treatment provided for in the experimental protocol, supernatants were replaced with 2% formaldehyde and left for 10 min for cell fixation. Fixed samples were visualized by transmitted light microscopy on Zeiss Axiovert 200 M Microscope (Zeiss, Göttingen, Germany) at 20× magnification and also with 100× oil immersion objective. ImageJ 1.54g; Java 1.8.0_345 [64-bit] software was used for image processing.

### 2.7. Statistics

Graphs generation and statistical analysis were performed using GraphPad Prism software version 10.2.1 for Windows. Results are presented as mean ± SEM. Differences with *p*-value of <0.05 were considered to be statistically significant. To quantify the synthesis of the 5-LOX product, a two-way ANOVA followed by Tukey’s multiple comparison test were used. For the statistical analysis of calcium dynamics, adhesive properties, and morphology, a one-way ANOVA test with Dunnett’s (for calcium assessment) or Tukey’s (for adhesion/morphology) multiple comparison tests were used.

## 3. Results

### 3.1. Extracellular ATP (eATP) Facilitated Formyl Peptide-Induced LT Synthesis

**Figure 1:**
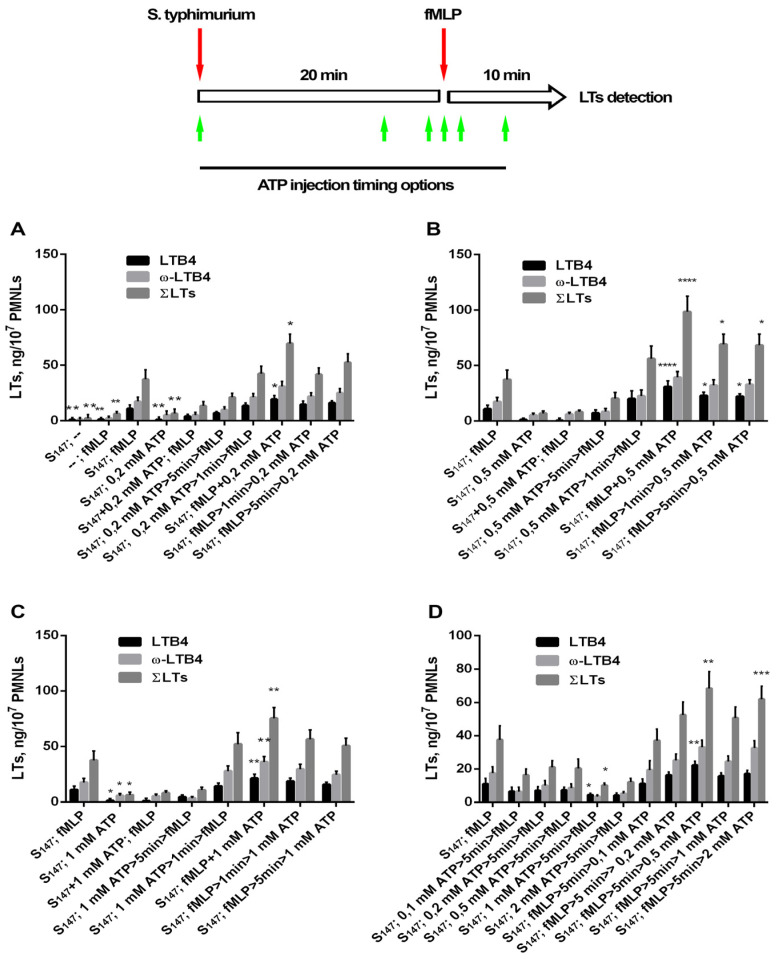
Effect of extracellular ATP on leukotriene synthesis in neutrophil interaction with the bacterium *Salmonella typhimurium*. The ratio of bacteria:PMNLs was ~25:1. The algorithm for two-stage PMNLs stimulation is schematically presented (top). The duration of incubation from the moment of bacteria addition to the moment of fMLP addition remained constant (20 min), as well as the interval from fMLP addition (0.1 µM) to incubation interruption (10 min). Timing options for ATP addition are presented (green arrows). The first treatment included bacteria (S_147_) or bacteria and ATP (S_147_ + ATP). The second treatment included either 10 min with ATP alone or a different addition sequence of fMLP (0.1 µM) alone (S_147_; fMLP—sample served as control) or fMLP combined with ATP (indicated concentrations): ATP before fMLP (ATP > time > fMLP), ATP together with fMLP (ATP + fMLP), and ATP after fMLP (fMLP > time > ATP). Data presented for 0.2 mM ATP (**A**), 0.5 mM ATP (**B**), 1 mM ATP (**C**), and for 0.1–2 mM ATP (**D**). Presented here are the absolute values of LTB4, ω-OH-LTB4, and the sum of LTs (ΣLTs). Values indicate mean ± SEM of three or more independent experiments performed in duplicates. * *p* < 0.05, ** *p* < 0.01, *** *p* < 0.001, **** *p* < 0.0001 for data compared to control by two-way ANOVA followed by Tukey’s multiple comparison test.

Earlier, we studied 5-LOX product synthesis in the experimental model of neutrophil interaction with the bacterium *Salmonella typhimurium* [35]. In support of previous results, it was shown that, in the absence of other stimuli, neutrophils culturing with non-opsonized bacteria was not accompanied by significant changes in arachidonic acid metabolism (Figure 1A), with fMLP alone being a weak inducer of leukotriene synthesis (Figure 1A). However, the interaction of neutrophils with bacteria strongly promoted leukotriene synthesis induced by subsequent addition of fMLP (Figure 1A). Addition of eATP as a secondary stimulus to neutrophils cultured in the presence of bacteria slightly increased the synthesis of leukotriene in comparison with bacteria alone (Figure 1A). Next, we investigated eATP influence on LTs production in an experimental algorithm involving sequential stimulation of cells with both bacteria and the chemoattractant fMLP. The main 5-LOX products in our experimental model were LTB4 and ω-OH-LTB4 [35]. We present data for LTB4, the omega-hydroxylation product of LTB4—ω-OH-LTB4 (ω-LTB4), and total leukotrienes ΣLTs (ΣLTs = LTB4 + isomers of LTB4 + ω-OH-LTB4).

Depending on the mode of ATP addition to the cells, we observed inhibition as well as stimulation of 5-LOX product formation. The LT synthesis in infected neutrophils was sensitive to the sequence of combined ATP and fMLP. eATP added together with bacteria, i.e., early (20 min) before fMLP, strongly inhibited fMLP-induced LT synthesis (Figure 1A–C). When added 5 min before fMLP, eATP inhibited LT synthesis in a concentration-dependent manner, the effect of which was stronger at higher concentrations of eATP (Figure 1D). eATP added simultaneously with fMLP increased LT synthesis (Figure 1). Addition of ATP 5 min after fMLP produced stronger responses with increasing ATP concentrations (Figure 1D). Figure 1D clearly shows the opposite effects of pre- and post-eATP-addition: when neutrophils encountered eATP before fMLP, ATP inhibited LT synthesis, while, when neutrophils encountered eATP after fMLP, ATP dose-dependently increased LT synthesis. Similar effects of pre- and post-eATP-addition were observed with opsonized bacteria (Supplementary Figure S1).

### 3.2. Bacteria and eATP Modulate Ca^2+^ Fluxes in Neutrophils Exposed to the Bacterium Salmonella typhimurium

**Figure 2:**
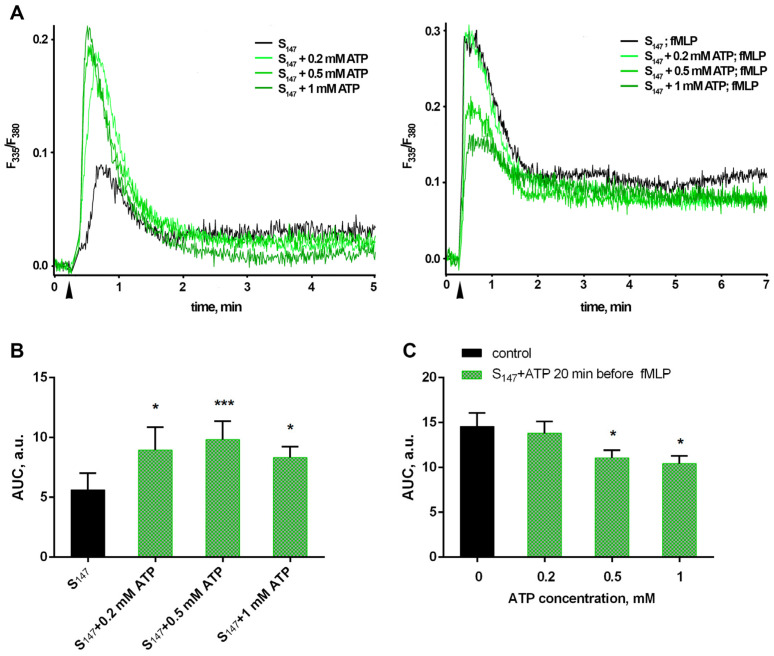
With sequential stimulation of neutrophils by the bacterium *S. typhimurium* and fMLP, the addition of ATP simultaneously with the bacteria affects the dynamics of [Ca^2+^]_I_ changes, including smoothing out the calcium stimulation effect of fMLP. Fura-2 AM-loaded PMNLs suspension in HBSS/HEPES (5 × 10^5^ cells/well) was kept for 5 min at 37 °C, 5% CO_2_. Then, *S. typhimurim* (S_147_) alone or in combination with 0.2–1 mM ATP (indicated) were injected. A total of 20 min later, 0.1 µM fMLP was added. Fluorescence intensities (335 nm/510 nm and 380 nm/510 nm) began to be recorded before each reagent injection, and measurements continued for 10 min after. (**A**) Typical curves of [Ca^2+^]_I_ changes (ratio F_335_/F_380_) when adding bacteria (black) or bacteria with ATP (green) (left) and with the subsequent fMLP stimulation (right) (arrows—injection time). (**B**) AUC (means ± SEM) for a two-minute interval after bacteria alone (black) or bacteria with 0.2–1 mM ATP (green) addition. (**C**) AUC (means ± SEM) for a two-minute interval after fMLP addition to PMNLs pretreated with bacteria only (black) or bacteria with 0.2–1 mM ATP (green). * *p* < 0.05, *** *p* < 0.001, for data compared to control by one-way ANOVA followed by Dunnett’s multiple comparisons test.

The ubiquitous second messenger, cytosolic Ca^2+^, plays an important role in the regulation of many cell functions, including leukotrienes synthesis. Translocation of key synthesis enzymes, cytosolic phospholipase A2 (cPLA_2_) and 5-LOX, to the nuclear envelope, necessary for both their activation and functionality maintenance, requires an increase in the intracellular free Ca^2+^ concentration ([Ca^2+^]_i_) [38,39,40]. Consecutive stimulation of neutrophils by the bacterium *S. typhimurium* and fMLP is accompanied by a biphasic increase in [Ca^2+^]_i_. With each influence, an early transient increase is followed by a prolonged persistence of calcium concentrations elevated above baseline (resting) levels over a long period of time (at least 20 min after bacterial stimulation and 10 min after the addition of fMLP). Both the amplitude of the rapid early increase and the level of persistent [Ca^2+^]_I_ elevation evoked by fMLP were markedly greater compared to those induced by bacteria (Figure 2A black curves). It seems that both periods of [Ca^2+^]_i_ increase are important for the assembly and optimal functioning of the cPLA_2_/FLAP/5-LOX enzyme group. As already noted, neither non-opsonized bacteria nor fMLP alone were able to induce a significant increase in leukotriene production.

Exogenous ATP causes elevation of intracellular calcium in neutrophils [41] (Supplementary Figure S2). Although the dynamics of the increase in [Ca^2+^]_i_ with the addition of ATP in the used concentration range is comparable to the effect of bacteria (Supplementary Figure S2), we note that subsequent treatment of PMNLs with ATP and fMLP just slightly potentiated LT synthesis (Figure 1). Addition of 0.2–1 mM eATP simultaneously with the bacteria increased the fulminant [Ca^2+^]_i_ rise compared to *S. typhimurium* alone (Figure 2A, green curves; 2B), but dose-dependently reduced the amplitude of the transient jump with further fMLP addition (Figure 2A, green curves; 2C). It can be assumed that the dynamics of the changes in [Ca^2+^]_i_ upon fMLP treatment play a decisive role in LT synthesis. Addition of ATP along with the bacteria prevents maximum calcium influx in response to fMLP, which affects the synthesis of leukotrienes (Figure 1A).

**Figure 3:**
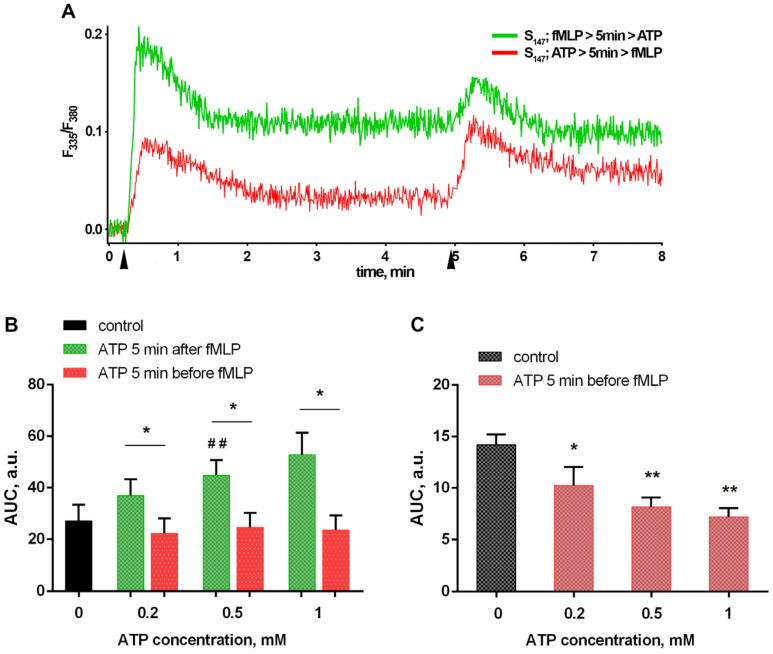
The dynamics of changes in [Ca^2+^]_i_ during the combined stimulation of ATP and fMLP depends on the sequence of stimuli application. Fura-2 AM-loaded PMNLs suspension in HBSS/HEPES (5 × 10^5^ cells/well) was kept for 5 min at 37 °C, 5% CO_2_. Then, the bacterium *S. typhimurim* (S_147_) was injected. Then, the cells were additionally stimulated with either fMLP or a combination of fMLP and ATP, adding them at 5 min intervals in different sequences. Fluorescence intensities (335 nm/510 nm and 380 nm/510 nm) began to be recorded before the second stimulation, and measurements continued for 10 min. (**A**) Typical [Ca^2+^]_i_ curves upon addition of fMLP 5 min before (green) or 5 min after 0.5 mM ATP (red) (arrows—injection time). (**B**) Total Ca^2+^ influx strengths in AUC (means ± SEM) for an eight-minute interval after an initial addition of fMLP (black, green) or ATP in the indicated concentrations (red). (**C**) AUC (means ± SEM) for a two-minute interval after fMLP stimulation of cells cultured in the presence of bacteria (black) or 5 min after the additional exposure to 0.2–1 mM ATP (red). * *p* < 0.05, ** *p* < 0.01, ^##^ *p* < 0.01, for data compared to control, and for pair of data, as indicated, by one-way ANOVA followed by Dunnett’s multiple comparisons test.

When fMLP and ATP were combined for the second stimulation, the sequence of ATP and fMLP addition significantly influenced the dynamics of [Ca^2+^]_i_ changes (Figure 3A). The total influx of [Ca^2+^]_i_, calculated as the area under the curve, was significantly higher following the addition of ATP 5 min after fMLP compared with both the addition before and the stimulation with fMLP only (control) treatments (Figure 3B), due to the greater amplitude of fMLP-induced growth (Figure 3C) and due to an increase in the level of free Ca^2+^ persistence after the transient jump.

The data obtained correlate with the results of leukotriene synthesis detection, that is, a proven dose-dependent inhibitory effect of ATP when added before fMLP and a potentiating effect when added five minutes later (Figure 1D).

### 3.3. Purinergic ATP Signaling Influences fMLP-Induced LT Synthesis in Neutrophils Exposed to the Bacterium Salmonella typhimurium

**Figure 4:**
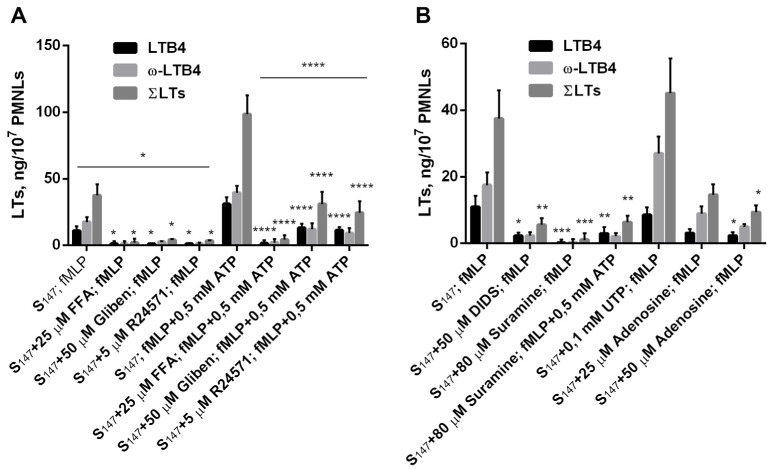
Interference with Ca^2+^ influx and purinergic signaling influence on fMLP-induced leukotriene synthesis in neutrophil interaction with the bacterium *Salmonella typhimurium*. Before treatment, PMNLs were preincubated for 10 min at 37 °C, 5% CO_2_. The first treatment (20 min) included bacteria (S_147_) or bacteria and indicated compounds: 25 µM FFA, 50 µM glibenclamide, 5 µM R24571 (**A**), 50 µM DIDS, 80 µM suramin, 100 µM UTP, 25 µM and 50 µM adenosine (**B**). The second treatment included 0.1 µM fMLP or fMLP and 500 µM ATP. Presented here are absolute values of LTB4, ω-OH-LTB4, and the sum of LTs (ΣLTs). Values indicate mean ± SEM of three independent experiments performed in duplicates. * *p* < 0.05, ** *p* < 0.01, *** *p* < 0.001, **** *p* < 0.0001 for data compared to corresponding controls by two-way ANOVA followed by Tukey’s multiple comparison test.

Neutrophil responses to chemoattractants are highly dependent on calcium (Ca^2+^) entry [4,42]. Potentiation of [Ca^2+^]_c_ increase under the influence of fMLP is achieved through the launch of several signaling pathways. In addition to its direct effect on the inositol triphosphate signaling cascade, leading to store-operated calcium channels opening [43], fMLP also promotes the opening of K^+^_ATP_ channels, which, due to cell hyperpolarization, augment the Ca^2+^ driving force through potential-dependent channels [44]. In addition, formyl peptides induce the release of ATP from PMNs [15] with a rapid extracellular ATP peak after 1 min of activation followed by very fast dissipation to control levels [45]. Neutrophils express pannexin (Panx 1) proteins, forming hemichannels that release ATP [18]. ATP further acts as an autocrine stimulator by binding to ligand-gated ion channels P2X7R. Figure 4A presents data on the sensitivity of fMLP-induced LT synthesis to several structurally diverse and selective inhibitors that block different aspects of Ca^2+^ signaling. Pannexin channel inhibitor [46] and store-operated calcium entry inhibitor flufenamic acid (FFA) blocked Ca^2+^ influx [47]. FFA inhibited fMLP-induced LT synthesis during neutrophil interaction with the bacterium *Salmonella typhimurium*, as well as in the presence of ATP (Figure 4A). The blocker of ATP-sensitive K^+^ channels glibenclamide, which is also known to block ATP efflux through connexin hemichannels [48], also inhibited LT synthesis. This effect was partially compensated for by the addition of eATP (Figure 4A). The impairment of calmodulin-operated mechanisms for opening the Ca channels [49] by the inhibitor of Ca^2+^/CaM complex R24571 [50] suppressed LT synthesis (Figure 4A).

Antagonists of purinergic ATP signaling inhibited fMLP-induced LT synthesis even in the absence of added eATP (Figure 4B). Panx 1 is sensitive to the anion channel inhibitor 4,4′- diisothiocyanostilbene-2,2′-disulfonic acid (DIDS) [51]. DIDS blocked the fMLP-induced release of ATP [18]. In our assay, DIDS inhibited fMLP-induced LT synthesis in PMNL interaction with the bacterium *Salmonella typhimurium* (Figure 4B), i.e., FPR responses required the release of cellular ATP. ATP is a P2X7 receptor ligand agonist [7]. The antagonist of the P2X7 receptor suramin inhibited LT synthesis (Figure 4B).

In healthy tissues, extracellular ATP released by cells is very rapidly dissipated to very low nanomolar concentrations due to plasma membrane-anchored ectonucleotidases. PMNLs express surface ectonucleotidases, CD39 (ectonucleoside triphosphate diphosphohydrolase-1), and CD73(ecto-5’-nucleotidase) that metabolize extracellular ATP into adenosine [17,52]. Neutrophils completely hydrolyzed exogenous ATP (5 mM) within 2 min after ATP addition, suggesting that they have potent ecto-ATPase activity [15]. Previously, it has been shown that ATP and UTP have similar effects on the intracellular Ca^2+^ concentration in *L.*-*donovani*-infected neutrophils [24]. In our assay, UTP, in contrast to ATP, did not inhibit 5-LOX product synthesis during early additions to the cells (Figure 4B). One can propose that ATP hydrolysis by ecto-ATPase and formation of adenosine can block 5-LOX activity during further stimulation with fMLP. Addition of adenosine to the cells suppressed fMLP-induced LT synthesis in our experimental model (Figure 4B).

### 3.4. eATP and Adhesive Properties of Neutrophils

**Figure 5:**
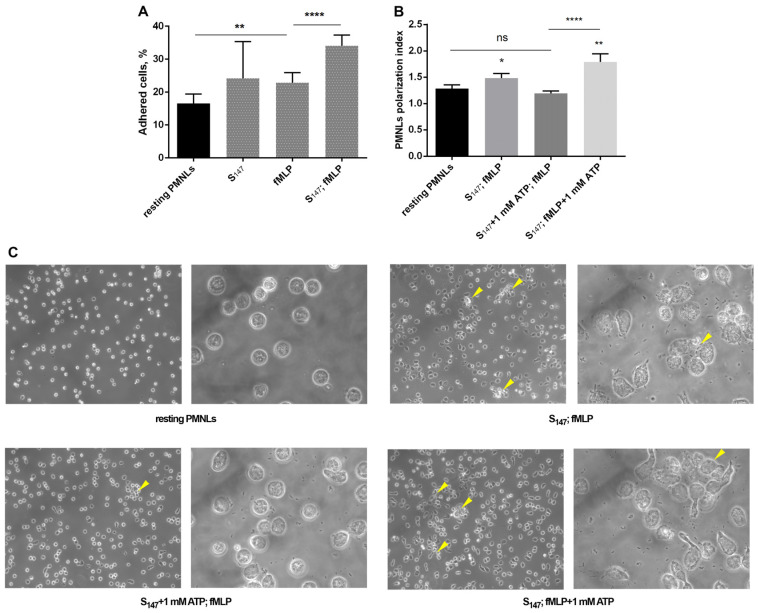
The effect of sequential treatment with bacteria and fMLP, as well as additional ATP stimulation, on adhesion (**A**) and morphology and clustering of neutrophils (**B**,**C**). (**A**) PMNLs were incubated for 20 min without additives (control, fMLP) or with *S. typhimurium* (S_147_, S_147_; fMLP). Then, 0.1 µM fMLP was added to the corresponding samples (fMLP, S_147_; fMLP). After 10 min, non-attached and weakly attached cells were removed from all samples and the proportion of adherent neutrophils was determined colorimetrically. The bars correspond to the number of attached PMNLs (% of the total); ** *p* < 0.01,**** *p* < 0.0001. (**B**,**C**) PMNLs were incubated for 30 min without additives (control) or with sequential stimulation by *S. typhimurium* (20 min) and 0.1 µM fMLP (10 min) (S_147_; fMLP). A total of 1 mM ATP was added simultaneously with either the bacteria or fMLP (indicated). The treated cells were fixed and visualized by transmitted light microscopy followed by image processing using ImageJ software. (**B**) Presented here are PMNLs polarization indices (means ± SEM; N = 3); * *p* < 0.05, ** *p* < 0.01 for data compared to control; ns—not significant, **** *p* < 0.0001 for pair of data indicated. (**C**) Representative images of cells obtained at 20× and 100× magnification. Arrows indicate cell clusters.

ATP is an active regulator of intercellular communication, which is important in neutrophil swarming. ATP release and the “calcium alarm” signal support the growth of dense antimicrobial neutrophil clusters [28]. ATP is a cell-aggregating substance and promotes intercellular contacts and Ca^2+^ fluxes between cells, promoting swarm progression. ATP promotes the activation and subsequent adhesion of neutrophils at sites of endothelial damage [53]. We analyzed the effect of ATP on changes in cell morphology under conditions of sequential stimulation with bacteria and fMLP. It was shown that, with a two-stage stimulation, substrate adhesion of neutrophils and cell polarity significantly increased (Figure 5A,B); in addition, microscopy analysis of samples at low magnification revealed heterogeneity in the distribution of stimulated cells on the substrate and a tendency to form clusters (Figure 5, arrows). Simultaneous addition of ATP and bacteria prevented both changes in cell morphology and the formation of clusters under the influence of fMLP (Figure 5B,C). When added simultaneously with fMLP, ATP supported the effect of fMLP on the polarization index and cluster formation (Figure 5B,C). These data correlate with data on the effect of ATP on leukotriene synthesis. It can be assumed that, under the conditions of interaction of neutrophils with bacteria, by potentiating or inhibiting fMLP-induced synthesis of leukotrienes, ATP contributes to the regulation of neutrophil swarming at sites of cell damage and pathogens invasion.

## 4. Discussion

The first immune cells to enter injured tissues are the neutrophils. They arrive in large numbers in response to DAMPs released from damaged and necrotic cells and orchestrate precise and efficient migration to a specific area of damage using a collective feed-forward mechanism called swarming [54].

Neutrophils also provide the first line of defense against pathogen invasions. High sensitivity to bacterial products is the main characteristic of neutrophils. This property is necessary for neutrophils to rush to sites of infection where these cells must destroy microbes to prevent their systemic spread. Formyl peptides of bacterial origin fuel leukotriene synthesis to stimulate neutrophil swarming [35]. Recently, with the discovery of swarming behavior by neutrophils, new activities of extracellular ATP have been found, connected with the propagation of the signals from the necrotic tissues [28]. The decisive role of Ca^2+^ and LTB4 has also been noticed [28]. The increase in eATP may influence the activation threshold of PMNLs. In our research, we focused on the impact of extracellular ATP on two interrelated processes: dynamic changes in intracellular [Ca^2+^] and LT synthesis in neutrophils interacting with the bacterium *Salmonella typhimurium*.

Increased plasma ATP levels have been demonstrated in both human sepsis clinical studies [55] and mouse sepsis models [56]. Infected or sterile lesions are also characterized by increased levels of extracellular ATP, which acts as a “danger signal” [57]. Both in the circulation and in the tissues, neutrophils are target cells for extracellular nucleotides. Increased levels of serum ATP contribute to the effective implementation of antimicrobial functions by neutrophils [58]. eATP, through P2X7 purinergic receptors, has been shown to promote the killing of bacteria in macrophages [59]. Extracellular ATP further promotes functional PMN responses [24]. CD11b expression, which is PMNLs activation marker, correlates with circulating plasma ATP levels [56]. It is known that LPS dramatically increases P2X and P2Y receptor expression, and bacteria injected into the abdominal cavity of mice have been found to stimulate ATP release from cells [60].

Necrotic and apoptotic cells release ATP, which can serve as a “find-me” signal [13]. However, the half-life of ATP is short, and additional signals are necessary to attract phagocytes over long distances [61,62]. Formylated peptides released from the mitochondria of damaged cells are an example of such an additional stimulus. They induce neutrophil activation and chemotaxis [63], and, together with ATP, guide phagocytes to the final encounter with target cells at inflammatory foci. Previously, it has been found that neutrophil phagocytosis is down-regulated by ATP, but this inhibiting action is completely lost in the presence of the bacterial products LPS or fMLP [64]. In our experimental model, the action of ATP was manifested in the presence of an “end-target”, the bacterial chemoattractant fMLP, and was realized during neutrophil interaction with the bacterium *Salmonella typhimurium*.

Formyl peptide receptors play an important role in pathogen recognition by PMNLs. FPRs stimulation triggers the production and release of ATP produced by mitochondria [18]. Further, autocrine stimulation by ATP influences PMNL functions [65]. Neutrophils use purinergic signaling as an antimicrobial host defense [66]. Bacterial LPS can trigger excessive mitochondrial ATP production and extracellular ATP release that disorganize neutrophil chemotaxis [66]. When bacteria use LPS for excessive ATP release from neutrophils, thus suppressing PMN chemotaxis, neutrophils rely on the enzymes ecto-ATPases that hydrolyze extracellular ATP, thus restoring chemotactic activity [66] and antimicrobial function [67].

Purinergic signaling was initially described by Geoffrey Burnstock in 1970 [68]. ATP is released from cells to activate purinergic receptors [69]. LPS and endotoxin-producing bacteria increase the affinity of P2X7 receptors for ATP [70]. ATP-induced P2X7 receptor activation causes a sustained increase in intracellular [Ca^2+^] and K^+^ efflux [20]. The stimulation of human PMNLs with ATP increases the expression levels of CD11b in PMNs [53]; CD11b expression is significantly reduced by ATP and ADP hydrolysis [71]. The ectonucleotidases CD39/ectonucleoside triphosphate diphosphohydrolase-1 and CD73/ecto-5’-nucleotidase are cell-surface enzymes that break down extracellular ATP into adenosine.

In our study, we found that eATP plays a significant role in the activation of LT synthesis in infected neutrophils. Maximal activation of LT synthesis was observed upon simultaneous addition of ATP and fMLP to neutrophils (Figure 1). Translocation of 5-LOX to the nuclear membrane is necessary for 5-LOX activity, as the latter requires the increase in intracellular calcium [72] and activation of mitogen-activated protein kinases (MAPK) [73]. fMLP itself stimulates both processes [42,74]; however, as we showed earlier, sequential stimulation with bacteria and fMLP is more efficient and resulted in 5-LOX translocation in almost every cell [35]. In this study, we found that eATP strengthened the effect of fMLP on LT synthesis by neutrophils interacting with the bacterium *Salmonella typhimurium* (Figure 1). Leukotriene synthesis potentiation appeared to correlate with increased [Ca^2+^]_i_ levels (Figure 1 and Figure 3). eATP is hydrolyzed by the enzymes on the surface of neutrophils and an early encounter with neutrophils, when fMLP is not available, results in processes that inhibit 5-LOX during further cell activation with fMLP. The experiments pointed to the role of ecto-ATPases, as well as to a possible role of ATP hydrolysis with the formation of adenosine, inhibiting LT synthesis (Figure 4). ATP addition to cells after fMLP resulted in a significant influx of Ca^2+^ (Figure 3), with further 5-LOX activation and increased LT synthesis (Figure 1D).

A positive feedback amplification mechanism for the attraction of neutrophils is mediated by the LTB4, which play a central role in neutrophil swarming [75]. It is especially important close to damaged and dying cells releasing not only ATP, but also mitochondrial N-formyl peptides disorganizing neutrophil chemotaxis by pathogens. The chemoattractant of host-cell origins, e.g., leukotriene B4 for positive-feedback-based recruitment of additional neutrophils, play a crucial role in such a situation. A sharp increase in the level of extracellular ATP in places subject to pathogen invasion, provided that formyl peptides of bacterial origin are present, can efficiently strengthen neutrophil response to bacteria and PAMPs. When neutrophils meet eATP without PAMPs, eATP was found to decrease leukotriene synthesis, thus terminating neutrophil swarming. In this way, eATP attract more neutrophils in cooperation with PAMPs to overcome pathogen invasion, but eATP decrease neutrophil influx to dead cells to smooth out inflammatory processes. These data identify a new mechanism for LT synthesis regulation in complex processes including PAMPs and DAMPs which must be taken into account in anti-inflammatory therapy.

## Data Availability

Data are contained within the article or Supplementary Materials.

## Supplementary Materials

The following supporting information can be downloaded at: https://www.mdpi.com/article/10.3390/biomedicines12061184/s1, Figure S1. 5-LOX product synthesis in human neutrophil (PMNLs samples (1.2–1.5) × 107/6 ml HBSS/HEPES) in incubations with opsonized bacteria *Salmonella typhimurium* (OS147) (the ratio of bacteria:PMNLs ~25:1). Bacteria were opsonized immediately before the experiment for 30 min in 20% (v/v) fresh serum from the same donor whose blood was used to isolate neutrophils; repeated centrifugation in Dulbecco’s PBS was used to wash the bacteria. Neutrophils were exposed to bacteria for 20min before adding of fMLP (0.1 μM) for next 10 min. Timing options for ATP adding were 5 min before fMLP, together with fMLP, and 5 min after fMLP. The 5-LOX products were analyzed using HPLC, and data LTB4, ω-OH-LTB4 and the sum of leukotrienes (ΣLTs = LTB4, iso-LTB4, ω-OH-LTB4) are presented. Values indicate mean ± SEM of three independent experiments performed in duplicate. * *p* < 0.05, ** *p* < 0.01, *** *p* < 0.001 for data compared to control by two-way ANOVA followed by Tukey’s multiple comparison test. Figure S2. eATP induces transient Ca^2+^-influx in neutrophils. Fura-2 AM-loaded PMNLs suspension in HBSS/HEPES (5 × 10^5^ cells/well) was kept for 5 min at 37 °C, 5% CO2. Then 0.2–1 mM ATP (indicated) or S. typhimurim (S147) were injected. Fluorescence intensities (335 nm/510 nm and 380 nm/510 nm) began to be recorded before each reagent injection, and measurements continued for 7 min after. (A). Typical curves of [Ca^2+^]I changes (ratio F335/F380) when adding ATP are presented. (B). AUC (means±SEM) for a two-minutes interval after bacteria (S147) or 0.2–1 mM ATP adding.
