# Supplementary material for: ATP and Formyl Peptides Facilitate Chemoattractant Leukotriene-B4 Synthesis and Drive Calcium Fluxes, Which May Contribute to Neutrophil Swarming at Sites of Cell Damage and Pathogens Invasion
Source: Biomedicines. 2024 May 27;12(6):1184. doi: 10.3390/biomedicines12061184 (PMC11201259; doi:10.3390/biomedicines12061184)
Supplement: Supplementary file 1 [file biomedicines-12-01184-s001.zip › biomedicines-2964885-supplementary.pdf]

*Article*

# ATP and formyl peptides facilitate chemoattractant leukotriene-B4 synthesis and drive calcium fluxes, which may contribute to neutrophil swarming at sites of cell damage and pathogens invasion


Ekaterina A. Golenkina[1], Galina M. Viryasova[1], Svetlana I. Galkina[1], Iuliia V. Iakushkina[1], Tatjana V. Gaponova[2], Yulia M. Romanova[3], and Galina F. Sud'ina[1]

[1] Belozersky Institute of Physico-Chemical Biology, Lomonosov Moscow State University, Moscow, Russia
[2] National Research Center for Hematology, Russia Federation Ministry of Public Health, Moscow, Russia
[3] Department of Genetics and Molecular Biology, Gamaleya National Research Centre of Epidemiology and Microbiology, Moscow, Russia


## Supplementary Figures

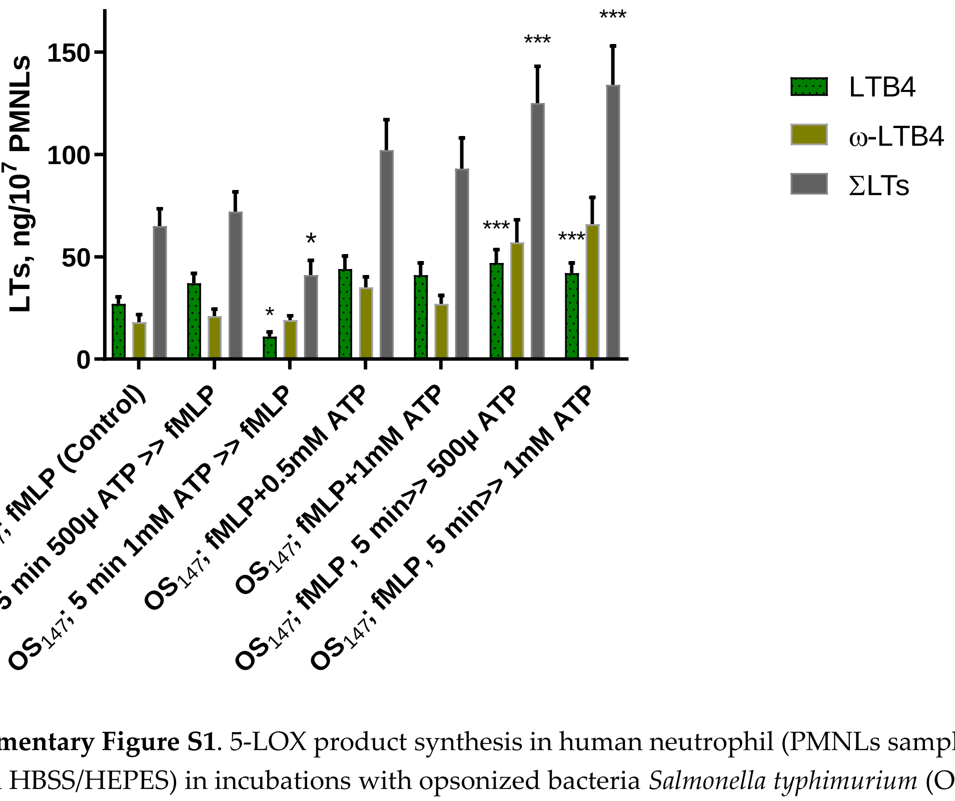


**Supplementary Figure S1**. 5-LOX product synthesis in human neutrophil (PMNLs samples (1.2–1.5) × $10^7$/6 ml HBSS/HEPES) in incubations with opsonized bacteria *Salmonella typhimurium* ($OS_{147}$) (the ratio of bacteria:PMNLs ~ 25:1). Bacteria were opsonized immediately before the experiment for 30 min in 20% (v/v) fresh serum from the same donor whose blood was used to isolate neutrophils; repeated centrifugation in Dulbecco's PBS was used to wash the bacteria. Neutrophils were exposed to bacteria for 20min before adding of fMLP (0.1 μM) for next 10 min. Timing options for ATP adding were 5 min before fMLP, together with fMLP, and 5 min after fMLP. The 5-LOX products were analyzed using HPLC, and data LTB4, ω-OH-LTB4 and the sum of leukotrienes (ΣLTs= LTB4, iso-LTB4, ω-OH-LTB4) are presented. Values indicate mean ± SEM of three independent experiments performed in duplicate. *$p < 0.05$, **$p < 0.01$, ***$p < 0.001$ for data compared to control by two-way ANOVA followed by Tukey's multiple comparison test.

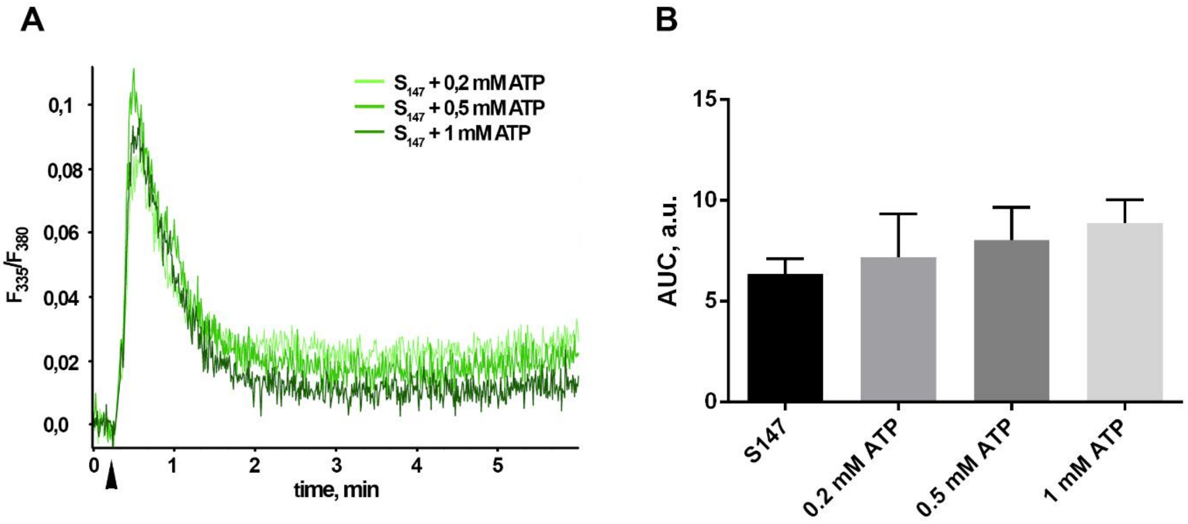


**Supplementary Figure S2**. eATP induces transient $Ca^{2+}$-influx in neutrophils. Fura-2 AM-loaded PMNLs suspension in HBSS/HEPES ($5*10^5$ cells/well) was kept for 5min at 37 °C, 5% $CO_2$. Then 0.2 – 1 mM ATP (indicated) or S. typhimurim ($S_{147}$) were injected. Fluorescence intensities (335 nm/510 nm and 380 nm/510 nm) began to be recorded before each reagent injection, and measurements continued for 7min after. **A.** Typical curves of $[Ca^{2+}]I$ changes (ratio F335/F380) when adding ATP are presented. **B.** AUC (means±SEM) for a two-minutes interval after bacteria (S147) or 0.2-1 mM ATP adding.
